# Fast Real-Time Model Predictive Control for a Ball-on-Plate Process

**DOI:** 10.3390/s21123959

**Published:** 2021-06-08

**Authors:** Krzysztof Zarzycki, Maciej Ławryńczuk

**Affiliations:** Faculty of Electronics and Information Technology, Institute of Control and Computation Engineering, Warsaw University of Technology, ul. Nowowiejska 15/19, 00-665 Warsaw, Poland; M.Lawrynczuk@ia.pw.edu.pl

**Keywords:** ball-on-plate process, model predictive control, PID controller, LQR controller, modeling

## Abstract

This work is concerned with an original ball-on-plate laboratory process. First, a simplified process model based on state–space process description is derived. Next, a fast state–space MPC algorithm is discussed. Its main advantage is computational simplicity: the manipulated variables are found on-line using explicit formulas with parameters calculated off-line; no real-time optimization is necessary. Software and hardware implementation details of the considered MPC algorithm using the STM32 microcontroller are presented. Tuning of the fast MPC algorithm is discussed. To show the efficacy of the MPC algorithm, it is compared with the classical PID and LQR controllers.

## 1. Introduction

Laboratories have a very important role in the successful education of engineering students. As far as automatic control is concerned, students use the classical laboratory processes such as the water tanks, the inverted pendulum, the magnetic levitation system and the ball-on-plate benchmark. In particular, the ball-on-plate process is very interesting in undergraduate and graduate courses since it requires multivariable stabilizing control [1,2,3]. Furthermore, it is a fast dynamical system that requires short sampling times of the controller. Because control of fast, unstable and multivariable systems is very important in different practical applications, the ball-on-plate process may be successfully used in control courses.

Since the ball-on-plate process may be described by state equations, Linear Quadratic Regulator (LQR) is frequently used [4,5,6]. Similarly, applications of the Sliding Mode Control (SMC) [5,7,8,9] and adaptive SMC [8,9] are reported. Furthermore, one may also try to use simple PD [2] or PID controllers [3,5,9,10]. More advanced solutions include fuzzy controllers [5,9,10], neuro-controllers [11], feedback linearization controllers [12] and disturbance-observer-based friction compensation schemes [13]. Finally, some works consider advanced Model Predictive Control (MPC) [14]. Historically, MPC algorithms have been used for industrial process control; example processes are chemical reactors [15], distillation columns [16], pasteurization plants [17] and fermentation systems [18]. Currently, MPC algorithms are also used for numerous other processes; example applications are: heating, ventilation and air conditioning systems [19], robotic manipulators [20], electromagnetic mills [21], servomotors [22], quadrotors [23], autonomous vehicles [24], unmanned aerial vehicles [25] and stochastic systems [26]. MPC algorithms have also been used for the considered ball-on-plate process. Unfortunately, the solutions presented in the literature are computationally demanding as they require on-line optimization carried out at each sampling instant. The MPC algorithm in which a nonlinear model is used for prediction and a nonlinear optimization task must be solved at each sampling instant on-line is presented in [27] (only simulations are shown). The MPC method based on a linearized model and quadratic optimization used on-line to calculate the manipulated variables is discussed in [28,29]. The objective of this work is to develop a fast MPC algorithm for the ball-on-plate process which does not need any on-line optimization and compare its performance with the classical PID and LQR controllers very frequently used for the considered process.

When the sampling time is short, e.g., of millisecond order, computational efficiency of MPC is an important issue [30]. The time required to perform calculations at every execution of the MPC algorithm must be shorter than the sampling time. There are a few methods that may be used to obtain short calculation time of MPC algorithms. First, specialized fast on-line optimization methods may be used, especially tailored for MPC applications [31]. Secondly, in the constrained explicit MPC algorithms [32], on-line optimization is not used, but a number of local explicit controllers are used. Successful implementation of the explicit MPC is reported [33], even when the available memory is limited [34]. Thirdly, the numerical optimization procedure used in the MPC algorithm may be replaced by a specially designed neural network which acts as a neural optimizer [35]. Fast explicit (analytical) MPC is possible in which the manipulated variables are calculated analytically using explicit formulas and next projected onto the admissible set determined by the constraints [36]. As a result, on-line optimization is not required. A practical application of this approach is described in [37], but only for processes described by simple step-response models and by discrete transfer functions (i.e., difference equations). This work follows the idea presented in [36,37] for state–space models. Finally, some specialized methods have been developed to handle constraints in on-line MPC optimization that make it possible to use sampling times of the order of milliseconds [16,38].

This work is concerned with an original ball-on-plate laboratory process. The contribution of this work is three-fold:

1. Simplified process modeling based on state–space process description is derived.
2. A fast state–space MPC algorithm is discussed and next applied to the considered ball-on-plate system. Its main advantage is computational simplicity: the manipulated variables are computed on-line using explicit formulas with parameters calculated off-line, no on-line optimization is necessary (nonlinear or quadratic). The presented state–space MPC algorithm uses a simple, yet very efficient state and output disturbance estimation necessary for prediction in state–space MPC [39].
3. Software and hardware implementation details of the MPC algorithm are presented.

The article is organized in the following way. Section 2 shortly describes the laboratory ball-on-plate process and introduces its model. The MPC algorithm is detailed in Section 3 while Section 4 deals with software and hardware implementation of the MPC algorithm using the STM32 microcontroller. Section 5 reports tuning of MPC and discusses results of experiments in which the discussed MPC scheme is compared with the classical PID and LQR controllers. Finally, Section 6 summarizes the whole article.

## 2. Ball-on-Plate Process

**Figure 1:**
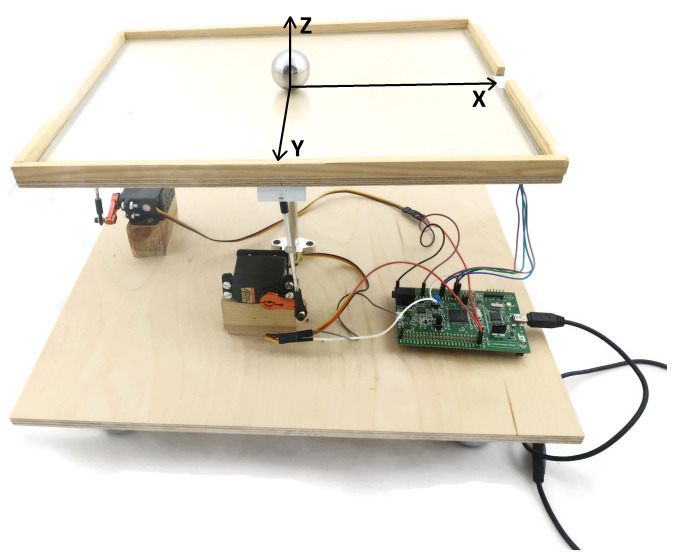
The ball-on-plate process.

This work is concerned with an original ball-on-plate laboratory system built at the Warsaw University of Technology and shown in Figure 1. Its construction details are given in [40]. In short, it includes: the touch pad, two digital servomotors, two servo arms and two servo rods, the microcontroller development board and some additional elements.

The process has two manipulated and two controlled variables. The Pulse Width Modulation (PWM) signals are used to control the digital servomotors. The plate rotates around the X and Y axes. Two signals that define the ball position on the top panel are the controlled variables. The actual ball position on the resistive touch panel is determined by two Analog to Digital Converters (ADCs). The STM32 F401C Disco microcontroller development board is used for implementation of control algorithms and communication with the PC computer (it is only used for saving the data and plotting the results).

Provided that friction and air resistance forces are neglected as well as angular velocities of the surface plate are low, the balance of the forces on the X axis is

(1) $$F_{\mathrm{tx}}\left(t\right)+F_{\mathrm{rx}}\left(t\right)=F_{\mathrm{gx}}\left(t\right).$$

Translation, rotation and gravity forces for the X axis are

(2) $$\begin{array}{cc} F_{\mathrm{tx}}\left(t\right) & =m{\ddot{x}}_{\mathrm{ball}}\left(t\right), \end{array}$$

(3) $$\begin{array}{cc} F_{\mathrm{rx}}\left(t\right) & =\frac{2}{5}m{\ddot{x}}_{\mathrm{ball}}\left(t\right), \end{array}$$

(4) $$\begin{array}{cc} F_{\mathrm{gx}}\left(t\right) & =mg\sin\Phi (t), \end{array}$$

where $x_{\mathrm{ball}}$ denotes the ball position along the X axis and Φ denotes the tilt angle of the plate in relation to the X axis. Similarly, for the Y axis, we have

(5) $$F_{\mathrm{ty}}\left(t\right)+F_{\mathrm{ry}}\left(t\right)=F_{\mathrm{gy}}\left(t\right),$$

where the forces are

(6) $$\begin{array}{cc} F_{\mathrm{ty}}\left(t\right) & =m{\ddot{y}}_{\mathrm{ball}}\left(t\right), \end{array}$$

(7) $$\begin{array}{cc} F_{\mathrm{ry}}\left(t\right) & =\frac{2}{5}m{\ddot{y}}_{\mathrm{ball}}\left(t\right), \end{array}$$

(8) $$\begin{array}{cc} F_{\mathrm{gy}}\left(t\right) & =mg\sin\Theta (t), \end{array}$$

where $y_{\mathrm{ball}}$ denotes the ball position along the Y axis and Θ denotes the tilt angle of the plate in relation to the Y axis. Using the forces (2)–(4) and (6)–(8), the model Equations (1) and (5) become

(9) $$\begin{array}{c} {\ddot{x}}_{\mathrm{ball}}\left(t\right)=\frac{5}{7}g\sin\Phi \left(t\right), \end{array}$$

(10) $$\begin{array}{c} {\ddot{y}}_{\mathrm{ball}}\left(t\right)=\frac{5}{7}g\sin\Theta \left(t\right). \end{array}$$

Let ϕ and θ denote rotation angles of the servo arm related to the Φ and Θ plate tilts, respectively. The relations are

(11) $$\begin{array}{cc} \sin\Phi (t) & =\frac{d}{L_{x}}\sin\phi \left(t\right), \end{array}$$

(12) $$\begin{array}{cc} \sin\Theta (t) & =\frac{d}{L_{y}}\sin\theta \left(t\right), \end{array}$$

where d=0.024 m is the servo arm length, $L_{x}=0.165$ m and $L_{y}=0.135$ m stand for distances between the point of attachment of the servo rod to the plate and the cross joint for X and Y axes, respectively. The values of *d*, $L_{x}$ and $L_{y}$ are constant and do not change in time. To remove nonlinearity, we use the approximations sinϕ≈ϕ, sinθ≈θ. It may be easily verified that for the angles $-20^{\circ }\leq \theta \;\leq 20^{\circ }$ and $-20^{\circ }\leq \phi \leq 20^{\circ }$, accuracy of the approximation used is very good. Using Equations (11) and (12), the model (9) and (10) becomes

(13) $$\begin{array}{c} {\ddot{x}}_{\mathrm{ball}}\left(t\right)=C_{x}\phi \left(t\right), \end{array}$$

(14) $$\begin{array}{c} {\ddot{y}}_{\mathrm{ball}}\left(t\right)=C_{y}\theta \left(t\right), \end{array}$$

where $C_{x}=\frac{5}{7}g\frac{d}{L_{x}}=1.0189$, $C_{y}=\frac{5}{7}g\frac{d}{L_{y}}=1.2453$. Equations (13) and (14) are expressed in the form of the classical linear continuous-time state–space model

(15) $$\begin{array}{cc} \dot{x}\left(t\right) & =A_{c}x\left(t\right)+B_{c}u\left(t\right), \end{array}$$

(16) $$\begin{array}{cc} y(t) & =C_{c}x\left(t\right), \end{array}$$

where the state, input and output vectors are

(17) $$x=\left[\begin{array}{c} x_{\mathrm{ball}}\\ {\dot{x}}_{\mathrm{ball}}\\ y_{\mathrm{ball}}\\ {\dot{y}}_{\mathrm{ball}} \end{array}\right],\;u=\left[\begin{array}{c} \phi \\ \theta \end{array}\right],\;y=\left[\begin{array}{c} x_{\mathrm{ball}}\\ y_{\mathrm{ball}} \end{array}\right].$$

The model matrices are

(18) $$A_{c}=\left[\begin{array}{cccc} 0 & 1 & 0 & 0\\ 0 & 0 & 0 & 0\\ 0 & 0 & 0 & 1\\ 0 & 0 & 0 & 0 \end{array}\right],\;B_{c}=\left[\begin{array}{cc} 0 & 0\\ C_{x} & 0\\ 0 & 0\\ 0 & C_{y} \end{array}\right],\;C_{c}=\left[\begin{array}{cccc} 1 & 0 & 0 & 0\\ 0 & 0 & 1 & 0 \end{array}\right].$$

The discrete-time version of the model (15) and (16) is

(19) $$\begin{array}{cc} x(k+1) & =Ax(k)+Bu(k), \end{array}$$

(20) $$\begin{array}{cc} y(k) & =Cx(k), \end{array}$$

where the model matrices are

(21) $$A=\left[\begin{array}{cccc} 1 & T_{s} & 0 & 0\\ 0 & 1 & 0 & 0\\ 0 & 0 & 1 & T_{s}\\ 0 & 0 & 0 & 1 \end{array}\right],\;B=\left[\begin{array}{cc} 0 & 0\\ T_{s}C_{x} & 0\\ 0 & 0\\ 0 & T_{s}C_{y} \end{array}\right],\;C=C_{c},$$

and $T_{s}$ denotes the sampling time.

## 3. Fast Model Predictive Control

Let $u={\left[u_{1}\;\ldots u_{n_{u}}\right]}^{T}$, $x={\left[x_{1}\;\ldots x_{n_{x}}\right]}^{T}$ and $y={\left[y_{1}\;\ldots y_{n_{y}}\right]}^{T}$ be the vector of process inputs (manipulated variables), the vector of states and the vector of outputs (controlled variables), respectively. For the considered process, $n_{u}=n_{y}=2$, $n_{x}=4$. At each sampling instant *k* of MPC, the following vector of increments is calculated [14]

(22) $$▵u\left(k\right)=\left[\begin{array}{c} ▵u(k|k)\\ \vdots \\ ▵u(k+N_{u}-1|k) \end{array}\right],$$

where $N_{u}$ is named the control horizon. The increments in Equation (22) are: ▵u(k|k)=u(k|k)−u(k−1) and ▵u(k+p|k)=u(k+p|k)−u(k+p−1|k) for $p=1,\ldots ,N_{u}-1$. The decision variables of MPC (22) are computed on-line as the result of minimization of the performance cost-function

(23) $$J\left(k\right)=\sum_{p=1}^{N}{∥y^{\mathrm{sp}}\left(k+p|k\right)-\hat{y}\left(k+p|k\right)∥}_{\Psi_{p}}^{2}+\sum_{p=0}^{N_{u}-1}{∥▵u(k+p|k)∥}_{\Lambda_{p}}^{2}.$$

The first part of the cost-function measures predicted control errors over the prediction horizon *N*. The vectors $y^{\mathrm{sp}}\left(k+p|k\right)$ and $\hat{y}\left(k+p|k\right)$ denote the required set-point of the controlled variables and the predicted vector, respectively, both for the sampling instant k+p and calculated at the instant *k*. The second part of the cost-function measures changes of the manipulated variables, which usually should be penalized. The weighting matrices $\Psi_{p}=\mathrm{diag}\left(\psi_{p,1},\ldots ,\psi_{p,n_{y}}\right)$ and $\Lambda_{p}=\mathrm{diag}\left(\lambda_{p,1},\ldots ,\lambda_{p,n_{u}}\right)$ are of dimensionality $n_{y}\times n_{y}$ and $n_{u}\times n_{u}$, respectively. Having computed the vector (22), only the increments for the current instant are applied to the process. The state and output process variables are then measured (or estimated) and the whole computational task is repeated at the next sampling instant.

As proved in [14], when the state–space linear model (19) and (20) is used for prediction, the predicted trajectory of the controlled variables, which is a vector of length $n_{y}N$

(24) $$\hat{y}\left(k\right)=\left[\begin{array}{c} \hat{y}\left(k+1|k\right)\\ \vdots \\ \hat{y}\left(k+N|k\right) \end{array}\right],$$

is given by the the following formula

(25) $$\hat{y}\left(k\right)=\tilde{C}M_{x}▵u\left(k\right)+{\tilde{I}}_{y}d\left(k\right)+\tilde{C}\left(\tilde{A}x\left(k\right)+V\left(Bu\left(k-1\right)+\nu \left(k\right)\right)\right),$$

where $\tilde{C}=\mathrm{diag}\left(C,\ldots ,C\right)$ is the matrix of dimensionality $n_{y}N\times n_{x}N$, the matrix of dimensionality $n_{x}N\times n_{x}N_{u}$ is

(26) $$M_{x}=\left[\begin{array}{ccc} B & \ldots & 0_{n_{x}\times n_{u}}\\ (A+I_{n_{x}\times n_{x}})B & \ldots & 0_{n_{x}\times n_{u}}\\ \vdots & \ddots & \vdots \\ \left(\sum_{i=1}^{N-1}A^{i}+I_{n_{x}\times n_{x}}\right)B & \ldots & \left(\sum_{i=1}^{N-N_{u}}A^{i}+I_{n_{x}\times n_{x}}\right)B \end{array}\right],$$

and the matrices of dimensionality $n_{x}N\times n_{x}$ are

(27) $$\tilde{A}=\left[\begin{array}{c} A\\ \vdots \\ A^{N} \end{array}\right],\;V=\left[\begin{array}{c} I_{n_{x}\times n_{x}}\\ \vdots \\ \sum_{i=1}^{N-1}A^{i}+I_{n_{x}\times n_{x}} \end{array}\right].$$

In this work, a very efficient state and output disturbance model introduced in [14] and thoroughly discussed in [39] is used. Conversely to the classical augmented state approach [41,42,43], it does not require an extension of the state vector. The state disturbance vector is computed as the difference between the measured or observed state vector, x(k), and the state variables calculated from the state model (19)

(28) ν(k)=x(k)−A(k)x(k−1)−B(k)u(k−1).

The output disturbance vector is computed as the difference between the measured output vector, y(k), and the corresponding values calculated from the output model (20)

(29) d(k)=y(k)−Cx(k).

The matrix ${\tilde{I}}_{y}={\left[I_{y}\ldots I_{y}\right]}^{T}$, where $I_{y}$ is the matrix of dimensionality $n_{y}N\times n_{y}$ whose all entries are 1. Using the predicted trajectory (25), the minimized MPC cost-function (23) may be rewritten in the following vector-matrix form

(30) $$\begin{array}{cc} J(k) & =∥y^{\mathrm{sp}}\left(k\right)-\tilde{C}M_{x}▵u\left(k\right)-{\tilde{I}}_{y}d\left(k\right)-\tilde{C}\left(\tilde{A}x\left(k\right)+V\left(Bu\left(k-1\right)+\nu \left(k\right)\right)\right){∥}_{\Psi }^{2}\\ \; & \;+{∥▵u(k)∥}_{\Lambda }^{2}, \end{array}$$

where the set-point trajectory is the vector of length $n_{y}N$

(31) $$\begin{array}{c} y^{\mathrm{sp}}\left(k\right)=\left[\begin{array}{c} y^{\mathrm{sp}}\left(k+1|k\right)\\ \vdots \\ y^{\mathrm{sp}}\left(k+N|k\right) \end{array}\right]. \end{array}$$

Typically, minimization of the MPC cost-function (30) is performed subject to constraints. The most useful ones include magnitude and rate of change of the manipulated variables

(32) $$\begin{array}{cc} \; & u^{\min}\leq u\left(k|k\right)\leq u^{\max}, \end{array}$$

(33) $$\begin{array}{cc} \; & ▵u^{\min}\leq ▵u\left(k|k\right)\leq ▵u^{\max}, \end{array}$$

where $u^{\min}$, $u^{\max}$, $▵u^{\min}$ and $▵u^{\max}$ are vectors of length $n_{u}$.

In this work, we derive a fast version of the state–space MPC controller [14]. A similar approach is discussed in [37] for processes described by simple discrete step-response models and difference equations. Minimization is performed without any constraints, but they are taken into account afterwards. Considering that the cost-function (30) is quadratic with respect to ▵u(k), the optimal increments of the manipulated variables for the current sampling instant are calculated analytically, without on-line optimization

(34) $$▵u\left(k|k\right)=K_{n_{u}}\left(y^{\mathrm{sp}}\left(k\right)-\tilde{C}\left(\tilde{A}x\left(k\right)+V\left(Bu\left(k-1\right)+\nu \left(k\right)\right)\right)-{\tilde{I}}_{y}d\left(k\right)\right),$$

where the matrix $K_{n_{u}}$ of dimensionality $n_{u}\times n_{y}N$ is

(35) $$K_{n_{u}}=\left[I_{n_{u}\times n_{u}}0_{n_{u}\times \left(n_{y}N-n_{u}\right)}\right]{\left(M_{x}^{T}{\tilde{C}}^{T}\Psi \tilde{C}M_{x}+\Lambda \right)}^{-1}M_{x}^{T}{\tilde{C}}^{T}\Psi .$$

Having calculated the unconstrained vector ▵u(k|k) from Equation (34), it is projected on the admissible set determined by the constraints (32) and (33) and the results are applied to the process. The projection procedure is

(36) $$\begin{array}{cc} \; & \mathrm{if}\;▵u\left(k|k\right)<▵u^{\min}\left(k|k\right)\;\mathrm{then}\;▵u\left(k|k\right)=▵u^{\min},\\ \; & \mathrm{if}\;▵u\left(k|k\right)>▵u^{\max}\;\mathrm{then}\;u\left(k|k\right)=▵u^{\max},\\ \; & u(k|k)=▵u(k|k)+u(k-1),\\ \; & \mathrm{if}\;u\left(k|k\right)<u^{\min}\;\mathrm{then}\;u_{n}\left(k|k\right)=u^{\min},\\ \; & \mathrm{if}\;u\left(k|k\right)>u^{\max}\;\mathrm{then}\;u_{n}\left(k|k\right)=u^{\max},\\ \; & u(k)=u(k|k). \end{array}$$

for manipulated variables, i.e., for $n=1,\ldots ,n_{u}$.

Let us stress the fact that the vector $K_{n_{u}}$ is calculated off-line for given model parameters and tuning coefficients of MPC. Hence, the explicit control law (34) depends only on precalculated parameters, the current set-point vector, estimations of the state and disturbance variables as well as on the value of the manipulated variables at the previous sampling instant.

## 4. Real-Time Implementation of MPC for Ball-on-Plate Process Using STM32 Microcontroller

### 4.1. Hardware Set-Up

In the experiments described below, the STM32 F401C Disco development board is used as the hardware platform for implementation of control algorithms. The considered development board offers all functionality required to develop a microprocessor-based embedded control system. Therefore, no other circuit boards are used in the system. The board uses the Cortex M4F core running at 84 MHz clocking and offers, among others: the Floating Point Unit (FPU), 256 kB flash memory and 64 kB SRAM memory. The microcontroller has a sufficiently large amount of RAM and the hardware FPU. The memory requirement is crucial since the MPC algorithm performs multiplication of matrices of large dimensionality. The STM32 F401C Disco contains all the I/O pins needed for the project.

The STM32 F401C Disco microcontroller development board is also responsible for communication with an external PC computer (using the serial port). This function is used only to send data later used to create plots presented in this work. All the calculations used in control algorithms are performed by the development board. Because of it, the floating point unit and a significant amount of memory are important features of the chosen microcontroller. The considered development board offers all necessary I/O pins, i.e., two PWM outputs to control the servomotors and two ADCs to read the ball’s position in two dimensions.

The digital Hi-tech HS 5485 HB [44] servomotors are used as the actuators. The choice has been motivated by the fact that servos do not require the use of an additional control loop for engine rotation. Additionally, their relatively low cost is also an essential advantage. The servomotors are equipped with a proportional controller. The Pulse Width Modulation (PWM) signal is used to control them, the pulse cycle is equal to 20 ms and pulse width is in the 900–2100 μs range. Their rotational range is $60^{\circ }$, the maximum speed is 0.2 $s/60^{\circ }$.

The Green Touch 15${}^{\prime }$${}^{\prime }$ 4-wire resistive screen pad is used [45] for detection of the ball location. In general, there are a few methods that may be used for touch detection: capacitive, infrared, surface acoustic wave and resistive. The resistive touch panel has been chosen because of its advantages: the ball may be made of any material, resistance to water and dust pollution and low cost. The main advantage, however, is the fact that the resistance touch screen is very easily controlled via the microcontroller’s pins. The ADCs simply read the current X and Y position of the ball. The ball position is determined by measuring horizontal and vertical resistances of the plate. The main drawback of the used sensor is the fact that the touch panel is very sensitive to disturbances. The pressure acting on it must be sufficient. The ball’s mass should be not lower than 70 g. If the force is too small, two different effects can be observed: no touch is detected and the sensor returns Not a Number (NaN) signal or the ball is temporarily detected in a different place, other than its actual location. If the ball is lying on the plane without motion, both effects occur very rarely. However, when the ball is constantly rolling on the surface of the plate, measurement errors appear quite often, even for balls that have a weight 100 g or more. It is due to the fact that ball sometimes bounces and breaks away from the plate surface for a short moment. In other moments the tilt of the plate is large enough to reduce the component of the gravity force perpendicular to the plate to a value that makes the pressure force acting on the screen not sufficient. The following filters are used to reduce the effects of the mentioned phenomena:

- A filter that finds out when the sensor stops detecting the ball. The touch panel then returns position values zero or NaN. When the filter detects such a value, it is ignored and the last meaningful number is taken as the current position value.
- A filter that finds out when the sensor detects an incorrect ball position. If the current position change is greater than a certain threshold, it is ignored.
- An arithmetical filter that collects *n* measurements of the ball position and calculates the ball position as:
  (37) $$x=\frac{\sum_{i}^{n}x_{i}}{n},\;\;\;y=\frac{\sum_{i}^{n}y_{i}}{n}.$$
  The ADCs of the microcontroller operate with much higher frequency than the developed controllers. Therefore, it is possible to collect more than 100 measurements in one control loop. The value n=50 has been chosen experimentally, as the smallest one that is able to eliminate all measurement errors. This filter is necessary for the system to work properly. Without it, the measurement errors destabilize all control algorithms.
- A median filter, which could be used as an alternative to arithmetical filter described above.
- Additionally, a Kalman filter has been implemented in the system. Its main role is to serve as an observer that estimates the unmeasured ball velocity value later used in the MPC algorithm. However, during the experiments it was observed that using Kalman filter’s estimates of the ball position instead of the true position measurements, helps to improve the quality of control slightly, due to elimination of small interferences that slipped through the arithmetical/median filter. In other words, the estimated position signal is slightly smoother than the measured signal.

Width of the panel is 322 mm, its height is 247 mm, which determines the size of the whole process.

The pins of the microcontroller used to connect it to sensors and actuators are:

- pin PB10 is configured as TIM3 timer’s channel 3 and used to send PWM control signal to the X axis servomotor,
- pin PA1 is configured as TIM3 timer’s channel 2 and used to send PWM control signal to the Y axis servomotor,
- pins PA2, PA3, PB0, PB1 are connected to the resistive touch panel, their configuration change in time as described in Table 1.

### 4.2. Software System

The software system offers implementation of three control algorithms: PID, LQR, MPC. The code is written in the C programming language. Keli μVision 5.0 is used as a programming environment. Additionally, other tools are used to simplify programming tasks: STMCube MX to configure the microcontroller’s clocks frequencies and I/O pins, the STMStudio to transfer data between the microcontroller and an external PC, MATLAB to find the controllers’ settings.

An external PC computer is used only to archive and visualize various signals and data. All calculations are performed using the microcontroller. Matrix and vector multiplications are the most computationally demanding. The size of the matrices in the MPC controller is correlated with the sampling time. Therefore, it is important to choose an adequate sampling period. A short sampling time provides theoretically better control but requires long horizons and a lot of calculations whereas a longer one allows using shorter horizons and results in a less computationally demanding solution, but the control quality may be insufficient. Both prediction and control horizons have an impact on the dimensionality of vectors and matrices, the latter one determines the number of calculated decision variables.

**Figure 2:**
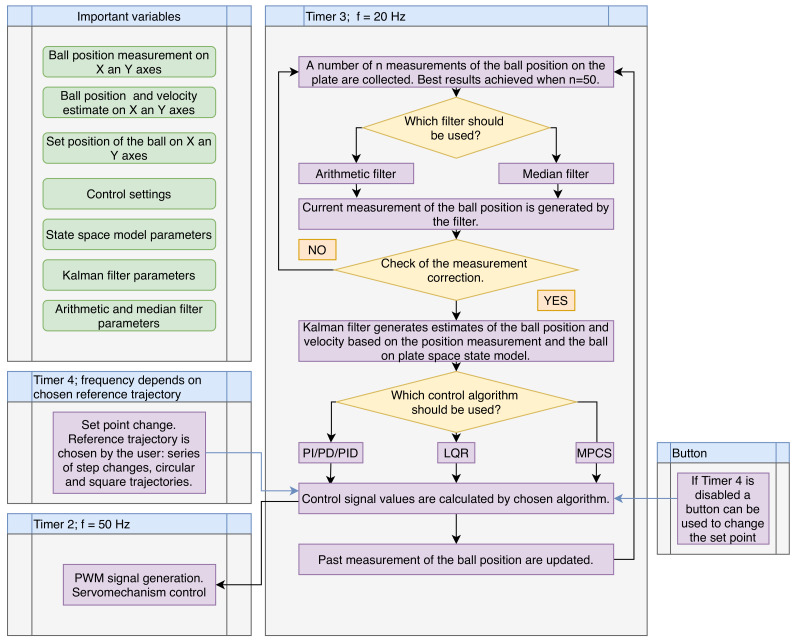
The structure of the real-time software system.

The structure of the real-time software system is depicted in Figure 2. It uses three timers. Discrete-time control algorithms must work in a strict time regime. The sampling time is 50 ms. Timer 3 is therefore set to work with the frequency of 20 Hz. As described earlier, the ball position sensor tends to produce many measurement errors. At each cycle, the timer routine begins with collecting *n* measurements of ball position from the touch panel via the ADCs of the microcontroller. The converters are sampled with higher frequency; therefore, during the 50 ms of the control loop, many measurements can be collected. Depending on the user’s preference, the arithmetic filter or the median filter is then used to generate the filtered current position of the ball as described by Equation (37). The best results are achieved when n=50. For lower values of *n*, measurement errors are frequent. For larger values, the measurements take too much time and cannot be performed in one control loop cycle.

Sometimes, the sensor is not pressed with force high enough and a false ball position is obtained. An additional function checks if the current measurement does make sense in relation to past measurements. Furthermore, the Kalman filter is used to generate estimates of the ball position and velocity based on the filtered measurement as well as the model (19) and (20). The control algorithm chosen by the user generates new control values. Finally, the ball position measurements are updated.

The servomechanisms’ control signals have a specific frequency, typically 50 Hz. Therefore, timer 2 is used. Its only purpose is to send the PWM signals from two of its channels to servomechanisms with duty cycle calculated based upon the values generated by the chosen control algorithm.

Timer 4 changes the set position of the ball. Its frequency varies depending on the reference trajectory chosen by the user. If the timer is disabled, the user can change the set-points by pressing a button.

Listing 1 shows a fragment of the code in which the values of the manipulated variables for the current sampling instant are calculated (Equation (34)). It is important that all vectors and matrices related to the linear model are constant, they are calculated off-line, i.e., $K_{n_{u}}$, $\tilde{C}$, $\tilde{A}$, V and B. Only the set-point vector, $y^{\mathrm{sp}}\left(k\right)$, the current state estimation, x(k), the manipulated variables at the previous sampling instant, u(k−1), as well as the output and state disturbance estimations, d(k) and ν(k), respectively, are updated on-line. Next, the calculated decision variables are projected onto the admissible set, determined by the constraints. For this purpose the procedure characterized by Equation (36) is used.

**Listing 1:** Fragment of the code: calculation of the control signal for the MPC controller.

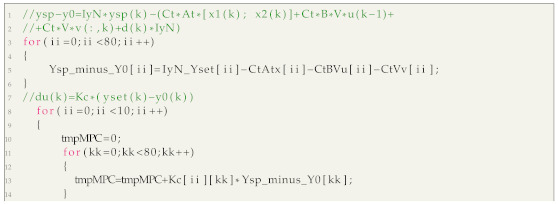



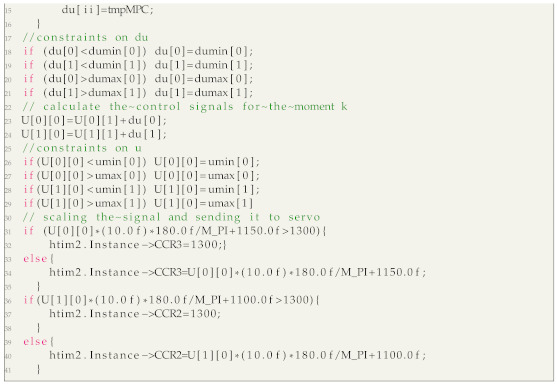



## 5. Results of Experiments

Three controllers have been implemented: MPC, PID and LQR. In all cases, the analytically calculated manipulated variables are projected onto the admissible set determined by the constraints

(38) $$\begin{array}{cc} \; & -20^{\circ }\leq \theta \;\leq 20^{\circ }, \end{array}$$

(39) $$\begin{array}{cc} \; & -20^{\circ }\leq \phi \leq 20^{\circ }. \end{array}$$

Tuning of the MPC controller starts with the selection of the prediction horizon. It should be long enough to cover the dynamic behavior of the process. However, if the horizons are too long, the matrices sizes rapidly increase as shown in Table 2, which results in longer computation time and memory requirement. The control horizon cannot be too short since it gives insufficient control quality while its lengthening increases the computational burden. The horizons N=40 and $N_{u}=5$ have been found experimentally. MATLAB simulations have been performed and next the horizons have been verified in real process. The process of tuning is the following:

- constant weighting coefficients $\lambda_{p,m}=1$ are assumed,
- the same prediction horizon *N* and control horizon $N_{u}$ are used; if the controller is not working properly, both horizons are lengthened,
- the prediction horizon is gradually shortened, and its length is chosen (with the condition $N_{u}=N$ ),
- the effect of the changing the length of the control horizon on the resulting control quality is assessed experimentally (e.g., assume successively $N_{u}=1,2,3,4,5,10,...,N$). The shortest possible control horizon is chosen.

**Figure 3:**
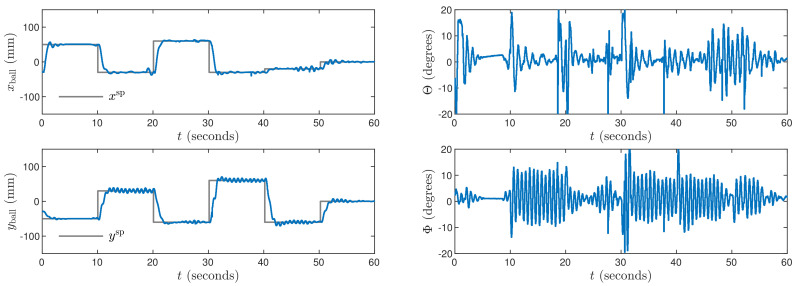
Experimental results of the MPC1 algorithm.

**Figure 4:**
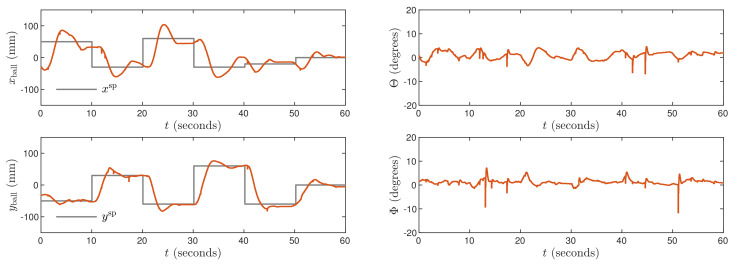
Experimental results of the MPC2 algorithm.

**Figure 5:**
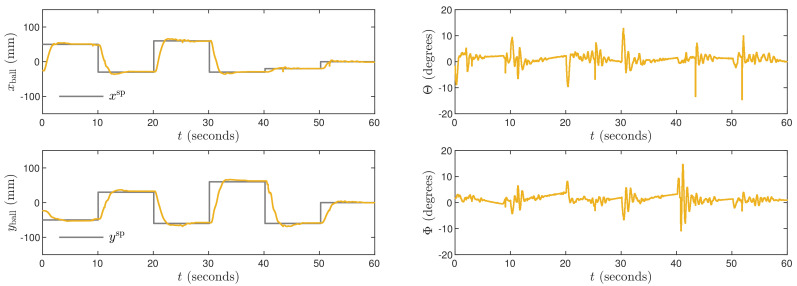
Experimental results of the MPC3 algorithm.

Next, the weighting coefficients must be tuned. Because the objective is precise control in both X and Y axes, $\psi_{p,n}=1$ for all p=1,…,N, $n=1,\ldots ,n_{y}$. Hence, the penalties $\lambda_{p,n}$ for all $p=0,\ldots ,N_{u}-1$, $n=1,\ldots ,n_{u}$ must be determined. Let us consider Figure 3 which shows trajectories obtained for the MPC1 algorithm with relatively small values $\lambda_{p,1}=0.35$ and $\lambda_{p,2}=1.1$. The process outputs very quickly follow changes of the set-points, but there are some small oscillations of the outputs and the manipulated variables change very rapidly. Let us consider Figure 4 that shows trajectories obtained for the MPC2 algorithm with relatively large values $\lambda_{p,1}=\lambda_{p,2}=500$. As a result, changes of the manipulated variables are very small, but control accuracy is insufficient, i.e., overshoot is high and the setting time is long. It can be noticed that the ball tends to stop its movement a few millimeters under or over the set-point. The mild changes in control signals are then unable to overcome the static friction force affecting the ball for a few seconds. After that time, the ball moves and stops on the other side of the set-point. This cycle then continues and as a result, the set position is never reached. After tuning, the best results are achieved for $\lambda_{p,1}=0.8$ and $\lambda_{p,2}=5$ in the MPC3 algorithm. The resulting process trajectories are depicted in Figure 5. In this case, the setting time is short, there are no oscillations and no overshoot.

**Figure 6:**
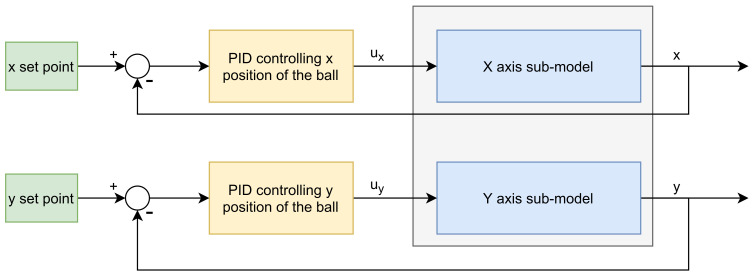
Diagram of the PID control structure for ball and plate system.

It is an interesting question whether the simple PID controller can give the control quality comparable with that possible when the well-tuned MPC3 scheme is used. The PID control system structure is shown in Figure 6. It consists of two independent classical single-loop PID controllers. The first of them controls the ball’s *x* position by manipulating the PWM signal of the first servo. The second one controls the *y* position by the PWM signal of the second servo. Two single-loop PID controllers may be used assuming that the interaction between the plate rotation around X axis and ball position *y* is negligible. The same applies for the rotation around Y axis and ball position *x*. For each sub-system, an independent PID controller has been tuned using the Ziegler–Nichols method and next readjusted experimentally. Let *K*, $T_{i}$ and $T_{d}$ denote the proportional gain as well as integration and derivation time constants, respectively.

**Figure 7:**
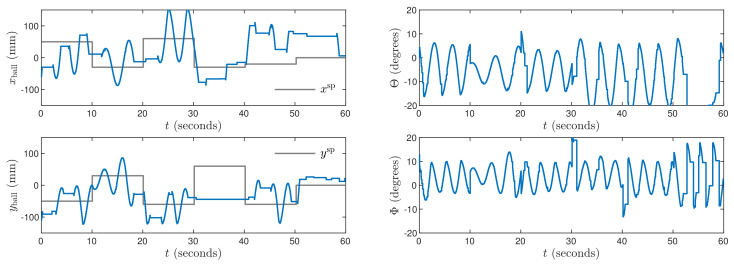
Experimental results of the PI algorithm.

**Figure 8:**
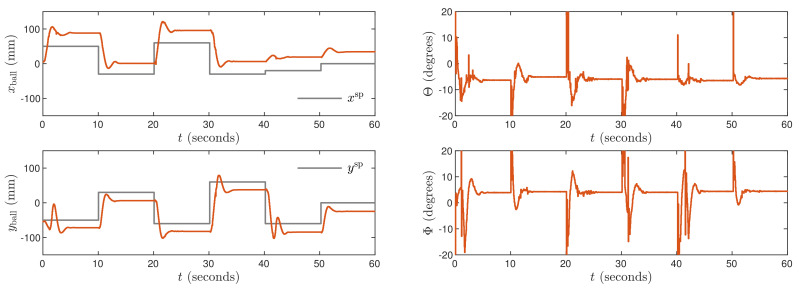
Experimental results of the PD algorithm.

**Figure 9:**
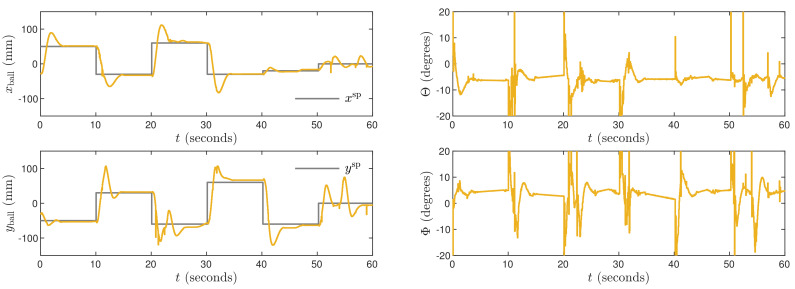
Experimental results of the PID algorithm.

The P controller is unable to stabilize of the system. Regardless of the used parameter *K*, the ball oscillates endlessly. Results for the PI controller are shown in Figure 7. Its tuning parameters are: $K_{x}=K_{y}=0.06$, $T_{\mathrm{ix}}=T_{\mathrm{iy}}=5$. It gives strong overshoot and slowly fading huge oscillations. The required set-points are never reached. Results for the PD controller are shown in Figure 8. Its parameters are: $K_{x}=0.09$, $K_{y}=0.07$, $T_{\mathrm{dx}}=T_{\mathrm{dy}}=0.6$. In comparison with the PI structure, it makes it possible to obtain smaller oscillations and reduced overshoot, but the steady-state error is inevitable. Finally, the PID controller is considered with the parameters: $K_{x}=0.09$, $K_{y}=0.08$, $T_{\mathrm{ix}}=T_{\mathrm{iy}}=1.5$, $T_{\mathrm{dx}}=T_{\mathrm{dy}}=0.6$. The obtained trajectories are shown in Figure 9. The PID controller makes it possible to eliminate the steady-state error. Unfortunately, let us stress the fact that the PID controller gives much worse control than the MPC3 scheme (Figure 5). In particular, the setting time is much longer, and significant overshoot is present.

PID and PD controllers give very rapidly changing control signals. If the calculations are performed using the ball position measurement, the derivative part causes the whole system to vibrate strongly, even when the ball reaches the set-points. This problem is eliminated using an estimate generated by the Kalman filter instead of the measurements.

The PID controller turns out to be very sensitive to measurement errors. Let us consider Figure 9 and t=55 s. A small measurement error causes the ball to move significantly away from the set-points, in particular for the $y_{\mathrm{ball}}$ output. Errors of the same order do not affect the MPC and LQR controllers.

Finally, the LQR controller is evaluated. Its settings are selected in two ways: (a) chosen experimentally using the pole placement method, (b) optimized in MATLAB using the DLGR function. For the poles 0.9404−0.0019i, 0.9404+0.0019i, 0.9404−0.0019i, 0.9404+0.0019i of the closed-loop system, the controller’s (LQR1) gain matrix is

(40) $$K_{1}=\left[\begin{array}{cccc} 1.3960 & 2.3049 & 0 & 0\\ 0 & 0 & 1.1422 & 1.8859 \end{array}\right].$$

In the first optimization approach (LQR2), the weighting matrices Q=diag(7,0,7,0), R=diag(1,1) give

(41) $$K_{2}=\left[\begin{array}{cccc} 2.4966 & 2.2137 & 0 & 0\\ 0 & 0 & 2.4813 & 1.9963 \end{array}\right].$$

In the second optimization approach (LQR3), the weighting matrices Q=diag(15,0,15,0), R=diag(1,1) give

(42) $$K_{2}=\left[\begin{array}{cccc} 3.6104 & 2.6621 & 0 & 0\\ 0 & 0 & 3.5837 & 2.3991 \end{array}\right].$$

**Figure 10:**
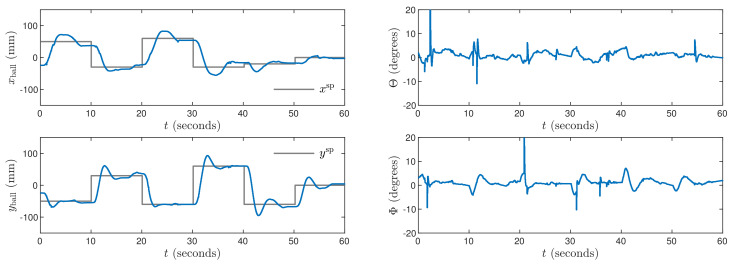
Experimental results of the LQR1 algorithm.

**Figure 11:**
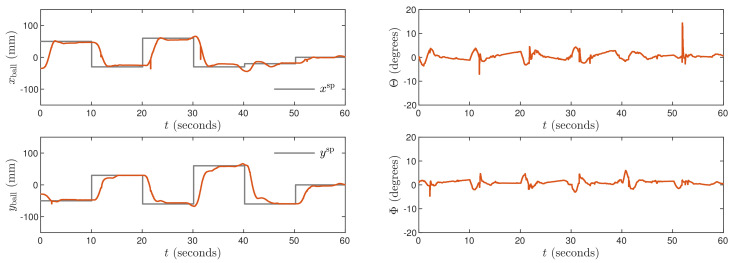
Experimental results of the LQR2 algorithm.

**Figure 12:**
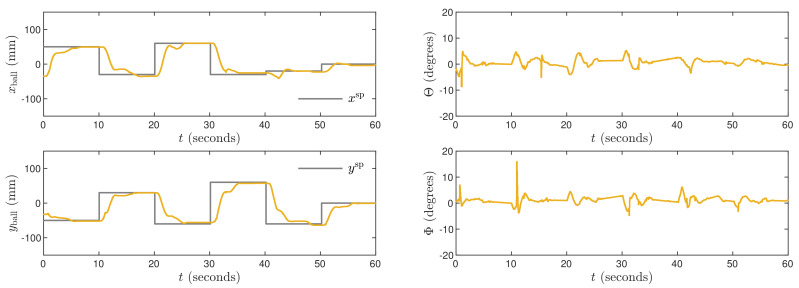
Experimental results of the LQR3 algorithm.

The obtained trajectories are given in Figure 10, Figure 11 and Figure 12 for the controllers LQR1, LQR2 and LQR3, respectively. In general, the manipulated variables change slowly. It is observed especially for the LQR2 and LQR3 controllers. No overshoot in a ball position signal is observed for those settings. However, the setting time is approximately two times longer than for the MPC3 algorithm (Figure 5). The LQR1 controller allows a shortening of that time, but overshoot appears. For the set-point tracking task, the LQR controllers perform much better than P, PD, PI, and PID ones, but still, the results are worse than in the case of the MPC3 scheme.

Let us compare all considered controllers numerically. Table 3 specifies control errors (the sum of squared control errors) [46] for all algorithms. The obtained values confirm observations possible on the basis on the process trajectories, i.e., the MPC3 algorithm is the best one. Table 4 specify the average and maximal setting times for all algorithms. Once again, the MPC3 algorithm gives the best results. Let us stress the fact that in the case of PI, PD, LQR1 and MPC2 controllers, it is impossible to determine the setting times for the assumed set-point changes, i.e., these control algorithms do not result is sufficiently fast control for the used changes of the set-points.

The calculation time for the explicit MPC control law (Equation (34)) and the projection procedure (Equation (36)) is 4.86 ms. Although it is longer than the time required by PID and LQR controllers, 0.29 ms and 0.27 ms, respectively, but it is important to point out that the obtained value is very short when compared to the sampling time used (50 ms).

## 6. Conclusions

This work discusses an application of the fast state–space MPC algorithm to the ball-on-plate laboratory process. Moreover, software and hardware implementation details are discussed; the STM32 microcontroller is used for calculations. The considered MPC algorithm is characterized by a very short calculation time (in comparison to the sampling period). This is because the manipulated variables are found on-line using explicit formulas with parameters calculated off-line, no real-time optimization is necessary. The simplicity of calculations is possible because in MPC an approximate state–space linear model is used, nonlinear terms are neglected. Secondly, the constraints are not taken into account during calculation of the manipulated variables, but afterwards, i.e., the unconstrained optimal solution is projected onto the admissible set determined by the constraints. When applied to the ball-on-plate process, the considered MPC algorithm works much better than the classical PID and LQR controllers. It is necessary to stress that all three controllers compared in this work are linear. The MPC and LQR controllers are multivariable, i.e., the manipulated variables are calculated taking into account all interactions of process variables and the same linear model is used for development of the control law. Conversely, the PID control scheme is comprised of two classical single-loop controllers.

The discussed MPC algorithm uses for prediction a simplified linear model of the process. In future, it is planned to develop nonlinear MPC approaches [47,48] and evaluate their performance.

## Figures and Tables

**Table 1:** STM32 F401C Disco pins configuration for communication with the touch panel.

Function	PA2	PA3	PB0	PB1
read x	output 1	ADC2	output 0	input no pullup
read y	ADC1	output 1	input no pullup	output 0

**Table 2:** Size of matrices used in MPC for different prediction and control horizons.

*N*	$N_{u}$	$\tilde{C}\tilde{A}$	$\tilde{C}VB$	*V*	$K_{n_{u}}$
20	3	40×4	40×2	80×4	6×40
40	5	80×4	80×2	160×4	10×80
80	10	160×4	160×2	320×4	20×160

**Table 3:** Control errors for all compared control algorithms (${\mathrm{ISE}}_{x}$: the error for the X axis, ${\mathrm{ISE}}_{y}$: the error for the Y axis).

Controller	${\mathrm{ISE}}_{x}$	${\mathrm{ISE}}_{y}$	${\mathrm{ISE}}_{x}+{\mathrm{ISE}}_{y}$
PI	7327.9	2759.2	10087.1
PD	1662.7	4420.9	6083.6
PID	483.9	837.9	1321.8
LQR1	692.3	825.6	1517.9
LQR2	684.3	793.7	1478.0
LQR3	676.0	849.9	1525.9
MPC1	881.5	498.0	1379.5
MPC2	1274.5	1378.6	2653.1
MPC3	373.7	760.0	1133.7

**Table 4:** Setting times for all compared control algorithms ($T_{x}^{\mathrm{avg}}$, $T_{x}^{\max}$: the average and maximal setting times for the X axis, respectively; $T_{y}^{\mathrm{avg}}$, $T_{y}^{\max}$: the average and maximal setting times for the Y axis, respectively).

Controller	$T_{x}^{\mathrm{avg}}$	$T_{x}^{\max}$	$T_{y}^{\mathrm{avg}}$	$T_{y}^{\max}$
PI	Impossible to determine			
PD	Impossible to determine			
PID	3.75	7	4.5	9
LQR1	Impossible to determine			
LQR2	4	7	5.5	8
LQR3	4.83	6	5.83	8
MPC1	3.17	4	5.17	7
MPC2	Impossible to determine			
MPC3	2.17	3	2	2

## Data Availability

The data presented in this study are available on request from the corresponding author.

## References

[1] Bay C.J., Rasmussen B.P. Exploring controls education: A re-configurable ball and plate platform kit. Proceedings of the 2016 American Control Conference (ACC).

[2] Fabregas E., Dormido-Canto S., Dormido S. (2017). Virtual and Remote Laboratory with the Ball and Plate System. IFAC-PapersOnLine.

[3] Stander D., Jiménez-Leudo S., Quijano N. Low-Cost “ball and Plate” design and implementation for learning control systems. Proceedings of the 2017 IEEE 3rd Colombian Conference on Automatic Control (CCAC).

[4] Dušek F., Honc D., Sharma K.R. Modelling of ball and plate system based on first principle model and optimal control. Proceedings of the 2017 21st International Conference on Process Control (PC).

[5] Kassem A., Haddad H., Albitar C. (2015). Commparison Between Different Methods of Control of Ball and Plate System with 6DOF Stewart Platform. IFAC-PapersOnLine.

[6] Spacek L., Bobal V., Vojtesek J. Digital control of Ball & Plate model using LQ controller. Proceedings of the 2017 21st International Conference on Process Control (PC).

[7] Bang H., Lee Y.S. (2018). Implementation of a Ball and Plate Control System Using Sliding Mode Control. IEEE Access.

[8] Jeon J., Hyun C. Adaptive sliding mode control of ball and plate systems for its practical application. Proceedings of the 2017 2nd International Conference on Control and Robotics Engineering (ICCRE).

[9] Morales L., Gordón M., Camacho O., Rosales A., Pozo D. A Comparative Analysis among Different Controllers Applied to the Experimental Ball and Plate System. Proceedings of the 2017 International Conference on Information Systems and Computer Science (INCISCOS).

[10] Robayo Betancourt F.I., Brand Alarcon S.M., Aristizabal Velasquez L.F. Fuzzy and PID controllers applied to ball and plate system. Proceedings of the 2019 IEEE 4th Colombian Conference on Automatic Control (CCAC).

[11] Moreno-Armendáriz M.A., Pérez-Olvera C.A., Rodríguez F.O. (2010). Indirect hierarchical FCMAC control for the ball and plate system. Neurocomputing.

[12] Huang W., Zhao Y., Ye Y., Xie W. State Feedback Control for Stabilization of the Ball and Plate System. Proceedings of the 2019 Chinese Control Conference (CCC).

[13] Wang Y., Sun M., Wang Z., Liu Z., Chen Z. (2014). A novel disturbance-observer based friction compensation scheme for ball and plate system. ISA Trans..

[14] Tatjewski P. (2014). Disturbance modeling and state estimation for offset-free predictive control with state-space models. Int. J. Appl. Math. Comput. Sci..

[15] Nebeluk R., Marusak P. (2020). Efficient MPC algorithms with variable trajectories of parameters weighting predicted control errors. Arch. Control Sci..

[16] Huyck B., De Brabanter J., De Moor B., Van Impe J.F., Logist F. (2014). Online model predictive control of industrial processes using low level control hardware: A pilot-scale distillation column case study. Control Eng. Pract..

[17] Pour F.K., Puig V., Ocampo-Martinez C. (2018). Multi-layer health-aware economic predictive control of a pasteurization pilot plant. Int. J. Appl. Math. Comput. Sci..

[18] Wang B., Shahzad M., Zhu X., Rehman K.U., Uddin S. (2020). A Non-linear Model Predictive Control Based on Grey-Wolf Optimization Using Least-Square Support Vector Machine for Product Concentration Control in l-Lysine Fermentation. Sensors.

[19] Carli R., Cavone G., Ben Othman S., Dotoli M. (2020). IoT Based Architecture for Model Predictive Control of HVAC Systems in Smart Buildings. Sensors.

[20] Rybus T., Seweryn K., Sąsiadek J.Z. (2018). Application of predictive control for manipulator mounted on a satellite. Arch. Control Sci..

[21] Ogonowski S., Bismor D., Ogonowski Z. (2020). Control of complex dynamic nonlinear loading process for electromagnetic mill. Arch. Control Sci..

[22] Horla D. (2021). Experimental Results on Actuator/Sensor Failures in Adaptive GPC Position Control. Actuators.

[23] Eskandarpour A., Sharf I. (2020). A constrained error-based MPC for path following of quadrotor with stability analysis. Nonlinear Dyn..

[24] Ducajú S., Salt Llobregat J.J., Cuenca Á., Tomizuka M. (2021). Autonomous Ground Vehicle Lane-Keeping LPV Model-Based Control: Dual-Rate State Estimation and Comparison of Different Real-Time Control Strategies. Sensors.

[25] Bassolillo S.R., D’Amato E., Notaro I., Blasi L., Mattei M. (2020). Decentralized Mesh-Based Model Predictive Control for Swarms of UAVs. Sensors.

[26] Bania P. (2020). An information based approach to stochastic control problems. Int. J. Appl. Math. Comput. Sci..

[27] Fan J., Han M. Nonliear model predictive control of ball-plate system based on gaussian particle swarm optimization. Proceedings of the 2012 IEEE Congress on Evolutionary Computation.

[28] Bang H., Lee Y.S. (2019). Embedded Model Predictive Control for Enhancing Tracking Performance of a Ball-and-Plate System. IEEE Access.

[29] Oravec M., Jadlovská A. Model Predictive Control of a Ball and Plate laboratory model. Proceedings of the 2015 IEEE 13th International Symposium on Applied Machine Intelligence and Informatics (SAMI).

[30] Houska B., Ferreau H.J., Diehl M. (2011). An auto-generated real-time iteration algorithm for nonlinear MPC in the microsecond range. Automatica.

[31] Wang Y., Boyd S. (2010). Fast model predictive control using online optimization. IEEE Trans. Control Syst. Technol..

[32] Bemporad A., Morari M., Dua V., Pistikopoulos E.N. (2002). The explicit linear quadratic regulator for constrained systems. Automatica.

[33] Valencia-Palomo G., Rossiter J.A. (2011). AEfficient suboptimal parametric solutions to predictive control for PLC applications. Control Eng. Pract..

[34] Rauová I., Valo R., Kvasnica M., Fikar M. Real-Time Model Predictive Control of a Fan Heater via PLC. Proceedings of the 18th International Conference on Process Control, Slovak University of Technology in Bratislava.

[35] Liu S., Wang J. (2006). A simplified dual neural network for quadratic programming with its KWTA application. IEEE Trans. Neural Netw..

[36] Tatjewski P. (2007). Advanced Control of Industrial Processes, Structures and Algorithms.

[37] Chaber P., Ławryńczuk M. (2019). Fast Analytical Model Predictive Controllers and Their Implementation for STM32 ARM Microcontroller. IEEE Trans. Ind. Inf..

[38] Valencia-Palomo G., Rossiter J.A. (2011). Programmable logic controller implementation of an auto-tuned predictive control based on minimal plant information. ISA Trans..

[39] Tatjewski P., Ławryńczuk M. (2020). Algorithms with state estimation in linear and nonlinear model predictive control. Comput. Chem. Eng..

[40] Zarzycki K., Ławryńczuk M., Bartoszewicz A., Kabziński J., Kacprzyk J. (2020). Development and modelling of a laboratory ball on plate process. Advanced, Contemporary Control.

[41] Maeder U., Morari M. (2010). Offset-free reference tracking with model predictive control. Automatica.

[42] Muske K., Badgwell T. (2002). Disturbance modeling for offset-free linear model predictive control. J. Process Control.

[43] Pannocchia G., Rawlings J. (2003). Disturbance models for offset-free model predictive control. AIChE J..

[44] HS-5485HB Standard Karbonite Digital Sport Servo. https://hitecrcd.com/products/servos/sport-servos/digital-sport-servos/hs-5485hb-standard-karbonite-digital-servo/product.

[45] 15” 4-Wire Resistive Screen. http://www.greentouch.com.tw/product/22-inch-four-wire-resistive-screen.html.

[46] Domański P. (2020). Control Performance Assessment: Theoretical Analyses and Industrial Practice.

[47] Ławryńczuk M. (2014). Computationally Efficient Model Predictive Control Algorithms: A Neural Network Approach.

[48] Marusak P.M. (2021). A numerically efficient fuzzy MPC algorithm with fast generation of the control signal. Int. J. Appl. Math. Comput. Sci..

